# Selections

**Published:** 1849-04-01

**Authors:** 


### Selections.

MEDICO-LEGAL INVESTIGATION.—F—— T——, 48 years of age, was sent last August to the lunatic asylum, at Fains, to be examined by a physician as to his sanity. He was a man below the middle size, of a nervous temperament, and weakly frame, the result, it is supposed, of constant poverty. Privations of all kinds had caused him to look much older than he really was, and the arms and head were affected with a continued slight trembling. He had, at times, some difficulty of breathing, and violent palpitations. The forehead was low, and depressed, marked with deep wrinkles, and covered with flattened hair.

The eyes were small and deep in the orbits, the cheekbones projecting, and the cheeks emaciated. The expression of the face showed but little intelligence, and the dull look, on being questioned, afforded additional proof of this opinion. He bore on the left side of the forehead the cicatrices of a seton, which had been made some time previously for a disease, the nature of which could not be ascertained. T—— was sent to the asylum by the authorities, in consequence of the plea of insanity urged by his advocates on his trial for murdering his master.

His antecedent history showed that he had been a labourer for many years, and had to support himself, wife, four children, and a mother-in-law, on a franc and a quarter (daily) wages. Penury accordingly weighed him down: badly lodged and ill-fed, he had no other resource to drive away care than by drinking brandy, in which he did not exceed. He did not know how to read or write, and did not carry his theology beyond the performance of his daily labour. In 1839, he robbed his master of a bag containing 600 francs, which he gave his wife. She returned it three days afterwards, but there were 120 francs missing. He denied the theft, and only confessed it lately before the *juge d'instruction*, averring at the same time that he had not abstracted any of the money, leaving it therefore to be inferred that his wife must have taken it. Ten years passed away after the commission of this crime, and he bore the character of being mild, and not quarrelsome, but rather irritable, and bearing unwillingly the jokes of his comrades. All the witnesses concurred in saying that he could have had no serious cause of hatred against his master; and he himself declared that his employer was very kind to him—quite a father.

M. Dagonet, by whom the case is published, tried at different examinations to ascertain the state of his mind, and he was led to believe that T—— was playing a part taught him by his advocate, although he did not appear to simulate insanity. It was easy to make him contradict himself. On one occasion, when pressed with questioning, he gave way to a convulsive laugh, which gave his features a bizarre and alarming expression. This was followed by a kind of dulness and depression, accompanied by tears and complaints of the misfortunes which continually pursued him. Early in September, the unhappy wretch strangled himself in bed, by passing a handkerchief round his throat, tying the two ends together, and then tightening it by a piece of wood, which he twisted round three or four times.

M. Dagonet expresses his opinion that T—— was a man of limited intellect, which had never been cultivated: a knowledge of what is right and feelings of honour had never been imparted to him. The theft was committed under a violent temptation, such as have before now influenced men in a less miserable condition than T——. His denial of the crime resulted from the fear of the prison—at all events, the fear of losing his situation. The murder was committed by him under an impulse of inconceivable rage, after a day's hard work, and after drinking some brandy. Had his intellectual faculties been more cultivated, the temptation might have been overcome. M. Dagonet considers the subsequent commission of suicide as caused by the sufferings of remorse. He does not regard him as affected with insanity, and consequently looked on him as amenable for his crime. Society, however, he observes, would not have had the right to demand a strict account, seeing that a well-directed education might have taught him to resist such passions, and that his intellects, naturally feeble, had been still further weakened by hard work and harder penury.—*Annales Medico-Psychologiques.*

Danger of too frequent Abstraction of Blood in the General Palsy of the Insane.—Notwithstanding many works have been published recently on the general palsy of the insane, its diagnosis and treatment are still surrounded with difficulties, even to those medical men who have paid especial attention to insanity, because there are but a few who are acquainted with the disease. It becomes, therefore, the duty of those who have made a special study of cerebral affections, to point out the difficulties which may be met with. Although, therefore, the fact is, that the information afforded by Dr. Lisle is not new, it is of importance, and so little known to the generality of practitioners, that he has conferred an obligation on the profession by its publication.

The variety of general palsy, in which Dr. Lisle especially points out the danger of bloodletting, is that form of the disease in which there occur, ordinarily without appreciable cause, general or partial epileptiform convulsions. These convulsions, which are more frequently followed by more or less marked prostration, which is almost always aggravated by repeated bleedings, resemble, to a certain extent, other affections of the nervous centres, which, in the majority of cases, imperatively require that mode of treatment, and with which, consequently, it is very important not to confound them. Dr. Lisle does not altogether condemn the abstraction of blood in general convulsive palsy; at the commencement of the disease, and in certain special conditions, he advises the application of leeches to the anus, or, better still, to the temple; but the remedy which, on the recommendation of M. Foville, he regards as truly heroic in the greater number of cases, is tartarized antimony, in large doses.—*Union Médicale.*

Suicides in France.—Dr. Dagonet remarks on the great increase in the number of suicides in France, consequent on the political troubles of the last year, which he attributes, in a great measure, to the impediments which resulted to commerce, the stagnation of affairs, and the consequent idleness, which, by depriving those who had the habit of working of their ordinary occupations, drove them into the sea of political agitation. Soon arises a passion, as it were, for the torrent which is drawing them on, and which, giving rise to powerful emotions, diminishes at the same time the normal impressionability, and renders them insensible to the pleasures of domestic life, which are soon altogether abandoned. Further, when the masses are roused by this universal fermentation, they soon give utterance to impious doctrines, which become the more attractive, from being apparently clothed in the garb of philanthropy, while they are often based on a frightful logic. The danger of striking at religion is equally great for society as for the individual. To represent the Deity as a myth, a sort of mask intended to alarm timid consciences; to attack all the feelings due to family and propriety, is at the same time to destroy the best instincts of man, to involve the mind which one pretends to illumine, in doubt rather than in light, and to lead subsequently to a contempt for mankind and the affairs of this world, which soon induces a tendency to suicide.—*Annales Medico-Psychologiques.*

Chloroform in Fractional Doses. M. Leriche has employed chloroform in many nervous affections—not as an anæsthetic, but as a powerful anodyne; he used it in combination with opium and other narcotics. His object being to relieve the pain without causing sleep, M. Leriche causes his patient to inhale a very small quantity, renewing the operation as soon as the pain returns. By these means, M. Leriche has succeeded in curing several patients; one of whom laboured under extremely violent nephritic colic; a second, under neuralgia of the cervical plexus; and the third, under dry asthma, which had not been relieved by either narcotics, camphor, or pharyngeal cauterization.—*Union Médicale.*

